# Using a generative co-design framework to adapt an exercise intervention as part of a multimodal intervention for patients’ receiving haemodialysis with or at risk of renal cachexia

**DOI:** 10.1186/s40900-026-00875-8

**Published:** 2026-04-17

**Authors:** Carolyn Blair, Adrian Slee, Clare McKeaveney, Alexander P. Maxwell, Faizan Awan, Malcolm Brown, Marion Carson, Sinead Comer, Andrew Davenport, Damian Fogarty, Denis Fouque, Oonagh Gooding, Teresa McKinley, Carolyn Hutchinson, Kamyar Kalantar-Zadeh, Karen Magee, Neal Morgan, Robert Mullan, Helen Noble, Sam Porter, David S. Seres, Joanne Shields, Ian Swaine, Miles D. Witham, Joanne Reid

**Affiliations:** 1 School of Nursing and Midwifery, Queen’s University Belfast, Belfast, UK; 2 Division of Medicine, Faculty of Medical Sciences, University College London, London, UK; 3 Centre for Public Health, Queen’s University Belfast, Belfast, UK; 4 Renal Patient Led Advisory Network (RPLAN), Lancashire, UK; 5 School of Sport and Exercise Science, Ulster University, Belfast, UK; 6 Renal Unit, Antrim Area Hospital, Northern Health and Social Care Trust, Antrim, UK; 7 UCL Department of Renal Medicine Royal Free Hospital, University College London, London, UK; 8 Regional Nephrology Unit, Belfast City Hospital, Belfast Health and Social Care Trust, Belfast, UK; 9 Division of Nephrology, Dialysis and Nutrition, Hôpital Lyon Sud and University of Lyon, Lyon, France; 10 Southern Health and Social Care Trust, Renal Unit, Daisy Hill Hospital, Newry, UK; 11 Irvine Division of Nephrology, Hypertension and Kidney Transplantation, University of California, Los Angeles, CA USA; 12 Department of Social Sciences and Social Work, Bournemouth University, Poole, UK; 13 Institute of Human Nutrition and Department of Medicine (Retired), Columbia University Irving Medical Center, New York, NY USA; 14 School of Human Sciences, University of Greenwich, London, UK; 15 AGE Research Group, NIHR Newcastle Biomedical Research Centre, Newcastle University, Newcastle Upon Tyne, UK

**Keywords:** Co-design, Cachexia, Exercise, Multimodal interventions, Haemodialysis, Muscle wasting

## Abstract

**Background:**

Currently there is insufficient evidence to inform the co-design of an exercise intervention as part of a multimodal intervention for renal cachexia. Co-design is an effective approach in collaborating with service users, carers and healthcare professionals to identify acceptable methods of improving delivery of care. The aim of this study was to use a co-design process to adapt an exercise intervention for patients with or at risk of renal cachexia as part of a cRCT for a multimodal intervention (NCT07107087)

**Methods:**

The objectives were as follows: (1) To co-design a strategy to promote optimal recruitment and adherence to an exercise intervention for those with or at risk of renal cachexia receiving HD, (2) To produce a conceptual model in relation to the implementation of an exercise intervention for this group. Using Bird and colleagues generative co-design framework for healthcare innovation, we adopted three stages of pre-design, co-design, and post-design. Accordingly, three workshops were conducted to correspond to each stage and the operational decisions recorded in seven steps to report the iterative design of the exercise intervention. The co-design workshops took place in November 2023 (*n* = 10), June 2024 (*n* = 11) and February 2025 (*n* = 6). Public co-design partners from Northern Ireland and England representing Kidney Care UK, Northern Ireland Kidney Patients Association and Northern Ireland Kidney Research Fund, participated in the workshops.

**Results:**

Contexts, intervention factors, mechanisms and outcomes which influence the uptake of, and adherence to, an exercise intervention within this patient population were identified. These included: the exercise intervention with an individualised and flexible approach; ensuring the exercise programme is manageable for patients receiving HD (session duration, timing and fistula awareness); ensuring the content of the exercise booklets is relatable and achievable (using household items rather than traditional exercise equipment and accrediting everyday activities as part of exercise log); providing support during the intervention (weekly telephone calls and progress tracking); and invitation to patients receiving HD considered most promising to encourage recruitment, sustain involvement and maximise impact from trusted healthcare professionals.

**Conclusion:**

Using the generative co-design framework for healthcare innovation, a conceptual model has been produced to promote optimal recruitment and adherence to an exercise intervention as part of a multimodal intervention for renal cachexia management in practice. This has informed component design, the wider implementation plan and evaluation design of a multimodal intervention for renal cachexia.

**Supplementary Information:**

The online version contains supplementary material available at 10.1186/s40900-026-00875-8.

## Abstract (plain language summary)

People living with kidney failure who receive haemodialysis (HD) can develop renal cachexia, a serious condition that causes muscle loss, weakness and poor quality of life. Exercise could help, but we do not yet know the best way to design and deliver an exercise programme that patients will find acceptable and manageable. Working with patients, carers and healthcare staff through co-design can help make sure any programme meets their needs.

**What we did**

We used a co-design approach to shape an exercise programme that will form part of a wider treatment package for people who have, or are at risk of, renal cachexia. Our goals were to:

1. Work with patients, carers and subject experts to design the best ways to invite people to join the programme and to help them stick with it.
2. Develop a clear model showing how an exercise programme for this group could be put into practice.

We followed a three-stage co-design framework (pre-design, co-design and post-design). Three workshops were held in 2023, 2024 and 2025 with public partners from Northern Ireland and England, including representatives from Kidney Care UK, the Northern Ireland Kidney Patients Association and the Northern Ireland Kidney Research Fund. We recorded decisions at each stage to guide the ongoing design of the programme.

**What we found**

Participants identified what helps, or makes it harder, for people receiving HD to take part in and continue with an exercise programme. Key points included:

- The programme must be personalised and flexible.
- Exercise sessions need to be manageable for people receiving HD, considering session length, timing and fistula (dialysis access site) safety.
- Exercise materials should feel achievable, using everyday items and recognising daily activities as exercise.
- Ongoing support, such as weekly phone calls and tracking progress, can encourage people to stay involved.
- Invitations to join the programme should come from trusted healthcare staff, which may improve recruitment and continued participation.

**What this means**

By using a co-design approach, we developed a clear model to support recruitment and adherence to an exercise programme for renal cachexia. The results directly shaped how the programme will be built, delivered and evaluated as part of a wider multimodal treatment approach.

**Supplementary Information:**

The online version contains supplementary material available at 10.1186/s40900-026-00875-8.

## Background

Pathological loss of muscle mass, or renal cachexia, is a major contributor to morbidity, excess mortality, increased healthcare costs and reduced quality of life [1]. For patients with kidney disease, protein-energy wasting (PEW) is deemed part of the continuum of cachexia and used interchangeably with cachexia or at risk of cachexia [2, 3]. In view of the strong association between adverse outcomes and the development of cachexia, authors have opined that patients with or at risk of cachexia should be supported to maintain body weight, improve strength, enhance the capacity for independent functioning and reduce frailty [4]. The pathogenesis of cachexia is believed to be multifactorial [5]. Multimodal interventions using exercise delivered adjuvant to conventional therapies are commonly recognised as the treatment of choice in cancer cachexia populations [6–8] and research on exercise and nutrition is building momentum in renal cachexia [9–13]. Given that aerobic and resistance exercise can increase the rate of metabolic stress and over time stimulate subcellular pathways involved in muscle protein synthesis [14], while also having a potential anti-inflammatory effect [15], exercise may help counteract syndrome-related muscle wasting [16]. Considering that patients with end-stage kidney disease receiving haemodialysis (HD) are most likely to experience the syndrome (75% of adults on dialysis show some evidence of muscle wasting) [17] this introduces complexity into the design of an exercise component as part of a multi-component intervention, due to the multifaceted ramifications of dialysis treatment.

The European Society for Medical Oncology (ESMO) guidelines emphasise the need for tailoring exercise to the stage of cancer cachexia and patient’s physical capacity and recommend resistance exercise two to three times per week as well as moderate aerobic (endurance) training [18]. This requires the expertise from a multidisciplinary team, as guidelines recommend that exercise prescription should involve a physiotherapist or an adequately trained professional [18]. The identified need for personalisation is similar in guidelines for CKD for example, Kidney Disease Outcomes Quality Initiative (KDIGO) guidelines [19] recommend that “*people with CKD undertake a cumulative duration of at least 150 minutes per week*” however this must be adapted to “*a level compatible with their cardiovascular and physical tolerance*.” In practice, this means that although HCPs are supportive of the benefits of exercise, they may under-prescribe or avoid advising about physical activity due to lack of CKD exercise/physical activity specialist staff [20, 21]. This points towards the need for adequate resourcing and education which helps to bridge this gap and support HCPs to deliver personalised exercise plans to their patients.

Although, there are reported benefits when using exercise as a unimodal treatment for cachexia management (e.g. increase in strength, function, muscle mass, and weight), there is no clear evidence that this is enough to target the multifactorial pathogenesis of cachexia [6–13, 22, 23]. Specific to renal cachexia, authors from small scale trials such as Hristea [24] have reported the feasibility and acceptability of a multimodal exercise and nutrition programme for PEW wherein partners completed a six-month training programme. As relevant to this co-design work, the exercise programme consisted of a progressive submaximal individualised cycling exercise using a cycloergometer during the dialysis session [24]. In cancer cachexia, the interim [25] larger scale trial (*n* = 221) of a multimodal intervention which used a behavioural home-based strength (resistance) and aerobic self-assisted exercise programme appeared promising in stabilising weight. High-quality trials of multimodal interventions using exercise for cachexia management are evidently in an infancy period, and there is little available evidence to relating to the optimum design of the components [7]. It is evident that achieving adequate statistical power and “clinically meaningful” changes in outcomes are resource-intensive goals; pilot and feasibility data are required for the confidence needed to design and carry out well-powered multimodal intervention randomised clinical trials (RCTs) [7].

The literature relating to exercise intervention for the general HD population is more advanced and can offer useful insight into the optimum design of an exercise component for this cohort. However, there is wide variation regarding exercise type, intensity, timing (e.g. intradialytic or home-based), safety precautions for patients receiving HD and an absence of directive guidelines [26]. A recent systematic review and meta-analysis focused on patients receiving HD, indicates that both intradialytic and home-based exercise types improved aerobic capacity, walking capacity, and health-related quality of life (HR-QoL) [27]. Evidence-based outcomes such as this are valuable as well as learning from co-design efforts which have sought to facilitate an exercise programme which best suits their public partners’ needs. Two of the most prominent and relevant exercise interventions which are suitable for people receiving HD are a digital health intervention to enhance physical activity in people with CKD (Kidney BEAM) [28] and intradialytic exercise program (COMEX) [29]. Both interventions [28, 29] incorporated patient involvement. The Kidney Beam online resource [28] was refined using feedback from people living with CKD based on their preliminary study as reported by Mayes and colleagues [30]. Similarly, in COMEX [29], feedback regarding design elements was incorporated from patients and dialysis staff and the intervention was then tested on two patient representatives. Although there has been an increase in the reporting of public involvement within health and social care research and many frameworks exist [31] rarely do studies use a framework for reporting such involvement [32]. 

Co-design is a methodology aiming to engage groups of stakeholders to aid in the development of interventions [33]. Co-design initiatives promote patient-centred care by prioritising the value of their lived experience in the design of the interventions [34]. It is recognised that realising the potential of research co-design requires more systematic reporting of the activities involved [35]. Bird and colleagues [32] generative co-design framework for healthcare innovation offers an approach which is closely with aligned with the Practical, Robust Implementation and Sustainability Model (PRISM) guidance [36] and is underpinned with the values of Participatory Action Research [37]; Community-Based Participatory Research [38]; and Experience-Based Co-Design [39]. Thus, this framework offers a methodical seven step process to engage partners meaningfully in the co-creation of the intervention and implementation strategies in order to fit local context and enhance equity. Aim:

To use a co-design process to adapt an exercise intervention for patients with or at risk of renal cachexia as part of a cRCT for a multimodal intervention (NCT07107087) [40].

## Methods

### Design

We used a co-design approach to engage with public co-design partners to aid in the development of this intervention through knowledge sharing [33, 41, 42]. This methodology was used to formally and meaningfully incorporate the ideas and values of our partners to ensure that this intervention was adapted to meet patient needs [43, 44]. This approach promoted patient-centred care by prioritising the value of our partner’s lived experience in the adaption of this exercise intervention while providing further insights than could have been gleaned from the research team alone [34].

**Fig. 1:**
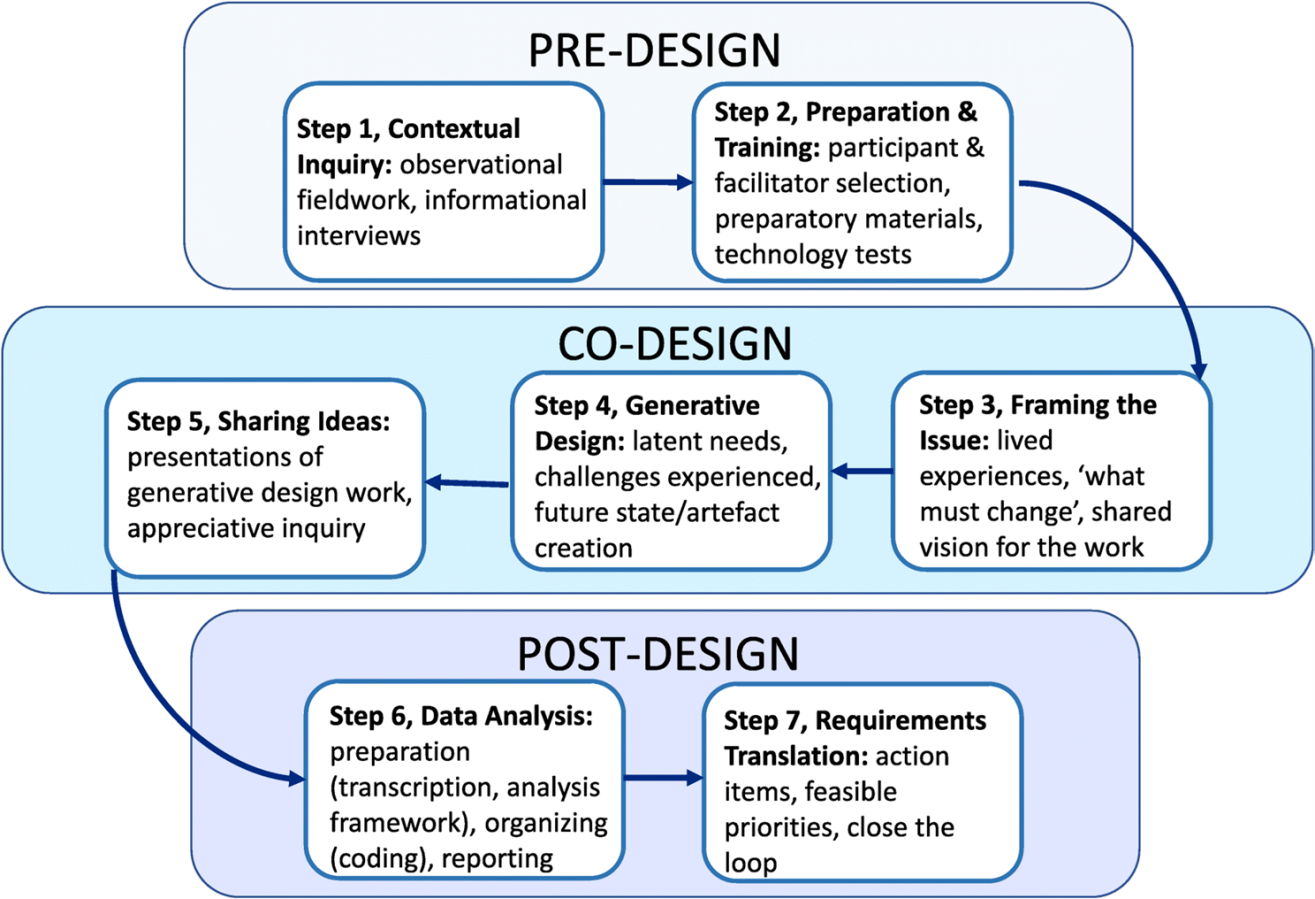
From: A generative co-design framework for healthcare innovation: development and application of an end-user engagement framework [28], figure is licenced under CC BY 4.0

Using Bird and colleagues [28] three stages of pre-design, co-design, and post-design three workshops were conducted and operational decisions made in each phase recorded to iteratively design the intervention. This Generative Framework for Healthcare Innovation uses a seven-step methodological process for co-design [32] which is structured in three stages comprising of seven steps. The seven-steps [28] (Fig. 1) detail the iterative process used to amend the design of an exercise intervention as part of a multimodal intervention for patients with or at risk of renal cachexia. These seven steps of development and application of the framework reciprocally informed each other.

### Objectives

1. To co-design a strategy to promote optimal recruitment and adherence to an exercise intervention for patients receiving HD with or at risk of renal cachexia.
2. To produce a conceptual model in relation to the implementation of an exercise intervention for this group.
3. To design an adapted handbook suitable for implementation as part of the multimodal intervention.

### Setting

Three co-design workshops took place in November 2023, June 2024, February 2025. All three workshops were held in-person in the United Kingdom.

### Participants

Eight public co-design partners with lived experience of receiving HD or being an informal caregiver for an individual receiving HD attended the first workshop along with three academic staff (see Supplementary document 1: Table 1). Six public co-design partners with lived experience of receiving HD or being an informal caregiver for an individual receiving HD attended the second workshop and five academic staff (see Supplementary document 1: Table 2). Three public co-design partners with lived experience of receiving HD or being an informal caregiver for an individual receiving HD attended the third workshop and three academic staff (see Supplementary document 1: Table 3). All public co-design partners received the National Institute of Health Research (NIHR) half day payment rate, which is reflective of their expert input and active involvement in this programme of research. For information regarding the quality, consistency and transparency of reporting we used the Guidance for Reporting Involvement of Patients and the Public 2 (GRIPP2) checklist (Supplementary document 2: GRIPP2).

### Analysis

The data analysis process was guided by the context-intervention-mechanism-outcome-impact (CIMOI) framework used widely in realist synthesis and evidence-based intervention design [45]. After applying the CIMOI coding framework the data generated in the workshops was reviewed to merge similar codes and identify the contexts, intervention factors, mechanisms and outcome themes in relation to how to successfully recruit to and implement an exercise intervention for patients receiving HD with or at risk of renal cachexia. The purpose of analysing the data which emerged from the co-design workshops was to identify the connections that workshop attendees made between features of the context and intervention and the resulting mechanisms and outcomes and hypothesise an impact.

## Results

What follows describes the iterative process to adapt an exercise-based intervention for patients receiving HD with or at risk of renal cachexia based the seven steps of Bird and colleagues [28] generative co-design framework for healthcare innovation.

### Pre-design

#### Step 1 – contextual inquiry

This step was focused on obtaining key stakeholder views on the potential development of a co-designed intervention for those with or at risk of renal cachexia. An initial workshop (Nov 2023) was held with stakeholders to discuss the prospect of a multimodal intervention for renal cachexia and particularly the components of the exercise intervention. The half-day workshop was hosted at a neutral venue based on the preferences of the public co-design partners external to the university to ensure utmost comfort and ease of transportation.

The facilitators (JR and CB) made suggestions to encourage an equal contribution from all members of the group with this and subsequent workshops. At the workshop there were two contextual talks to provide information about renal cachexia and the proposed multimodal intervention, each having structured discussion breaks. Subsequently, a more formalised feedback session was facilitated where partners were randomly divided into teams, each team of two/three started with one question and had 10 minutes to write responses (each having different coloured markers, flip chart paper and the ability to place a tick if they agree with what someone else’s view or an ‘x’ if not). After 10 minutes each team moved clockwise, with their uniquely coloured marker so as discussion post-session could easily be facilitated seeing the comments from each team. The four questions were based on gathering contextual thoughts about the specifications of 1) the exercise intervention; 2) psychosocial support; 3) the multimodal nature of the intervention, and 4) patient and caregiver education.

#### Step 2 – preparation and training

Based on the outcomes of the initial workshop, relevant materials were explored including COMEX [29] and Kidney Beam [28] and EXACT exercise intervention [46]. Despite the appeal of focusing on a digital intervention which has been designed for patients with CKD [28] or an HD specific programme [29], the research team deemed the EXACT exercise intervention [47] for patients with advanced cancer to be most suitable given the outcomes of the first workshop. The suitability for patients receiving HD was confirmed by the findings of the EXCITE trial (EXerCise Introduction to Enhance Performance in Dialysis) which showed that simple, personalised 6-month walking exercise intervention in patients on dialysis performed at home improved exercise capacity, as assessed by the 6MWT [47, 48]. Given the literature on combined resistance and aerobic activity the EXACT exercise intervention [46] consisting of 12-week home-based progressive, moderate-intensity walking and resistance exercise, two-five times per week [46] was deemed the most suitable. The rationale for the suitability of the EXACT intervention [46] was based on public co-design partners’ desire: to have ongoing in-person support, the flexibility to complete walking and resistance exercises consecutively or separately based on readiness (e.g. symptom burden) or preference, to have hard copies of an exercise handbook and exercise diary to monitor progress and for a home-based programme. The research team deemed this autoregulated programme suitable as it involves muscle strengthening and balance retraining exercises, progressing in difficulty over 12 weeks [46] which was the proposed length of the larger feasibility CRcT. Autoregulation refers to the permission of dose modifications to reduce the intensity of exercise and catch up on missed exercise based on individual readiness [46]. This was deemed particularly important by the public co-design partners who described the complexity of the treatment burden when receiving HD (e.g. fatigue after dialysis which was described as potentially lasting for 24 hours or longer) which was deemed similar to the original population who had a familiar experience post-chemotherapy. Furthermore, the EXACT intervention [46] was also deemed suitable as it had been successfully delivered with high retention to those who had similar functional ability to the expected cohort in a Multi-Modal Integrated intervention combining Exercise, Anti-inflammatory & Dietary advice (MMIEAD) who were unable or ineligible for high-intensity exercise.

### Co-design

#### Step 3 – sharing ideas

In the second workshop, two academics (IS and AS) with expertise in designing exercise interventions delivered two contextual talks. The contents were based on the evidence in relation to exercise in renal cachexia and fundamental principals in exercise interventions. Finally, an overview of EXACT [46] was provided. Using the same structure as the first workshop (step 1), partners were grouped into two/threes and presented with questions focused on 1) manageability for those receiving HD and if amendments are needed; 2) the resources and whether this is acceptable; 3) whether additional support or precautions are needed and who should deliver and, 4) who would be the best person to approach patients about recruitment to the intervention.

#### Step 4 – generative design

In the second part of the public involvement workshop, we collated the feedback from the interactive session and opened for further discussion. The outcome of the workshop confirmed that an amended version of the EXACT exercise programme specifically for those receiving HD would be acceptable as it had been successfully conducted with those with chronic illnesses including frail populations. It met the requirements for resistance and cardiovascular exercise through (1) a walking programme (2) a strengthening and toning programme. However, a series of suggestions were provided to adapt to programme to the HD population.

#### Step 5 – framing the issue

We took the findings from steps 1–4, compared and contrasted the EXACT intervention [46]. Additional requirements needed to be included in the intervention to encourage sustained involvement and maximum impact. These included, firstly the exercise intervention adopting a more individualised and flexible approach; ensuring the exercise programme is manageable for those receiving HD (fistula awareness, duration and timing). Secondly, ensuring the structure of the exercise booklets are relatable and achievable (using household items not traditional exercise equipment and crediting everyday activities as part of exercise log). Thirdly, support during the intervention (weekly telephone calls and progress tracking). Fourthly, invitation from consultant nephrologist, dietician or clinical research nurse to eligible patients (meeting pre-specified inclusion criteria) receiving HD. Fifthly and finally public co-design partners also suggested including an option for supervised, individualised intra-dialytic exercise should patients desire this as a supplement to the home-based programme. We then framed the findings of the workshops alongside the body of our research team’s reviews of the literature [5, 7, 8, 49] and wider relevant research to ensure contextual alignment with renal cachexia.

### Post-design

#### Step 6 - data analysis

To establish what works for the HD population, in which circumstances, through what mechanisms, and with what impacts we coded the important CIMOI factors using the responses to questions recorded on flip chart paper from workshop one and workshop two and agreed feedback to develop of a conceptual model for the exercise intervention, under consideration of context, effective intervention factors, leveraging specific mechanisms, targeting meaningful outcomes to optimise impact. Consideration was given to the most important aspects of the social and clinical context into which the intervention would be introduced. Firstly, the active components of the intervention. Secondly, the mechanisms for change embedded in these components. Thirdly, the outcomes that these mechanisms tend to generate in relation to the experience, knowledge and skills of partners in the intervention. Finally, the hypothesised sustained clinical impact that these outcomes may generate. These factors were drafted in consultation with key stakeholders (public co-design partners and research team).

#### Step 7 – requirements translation

**Fig. 2:**
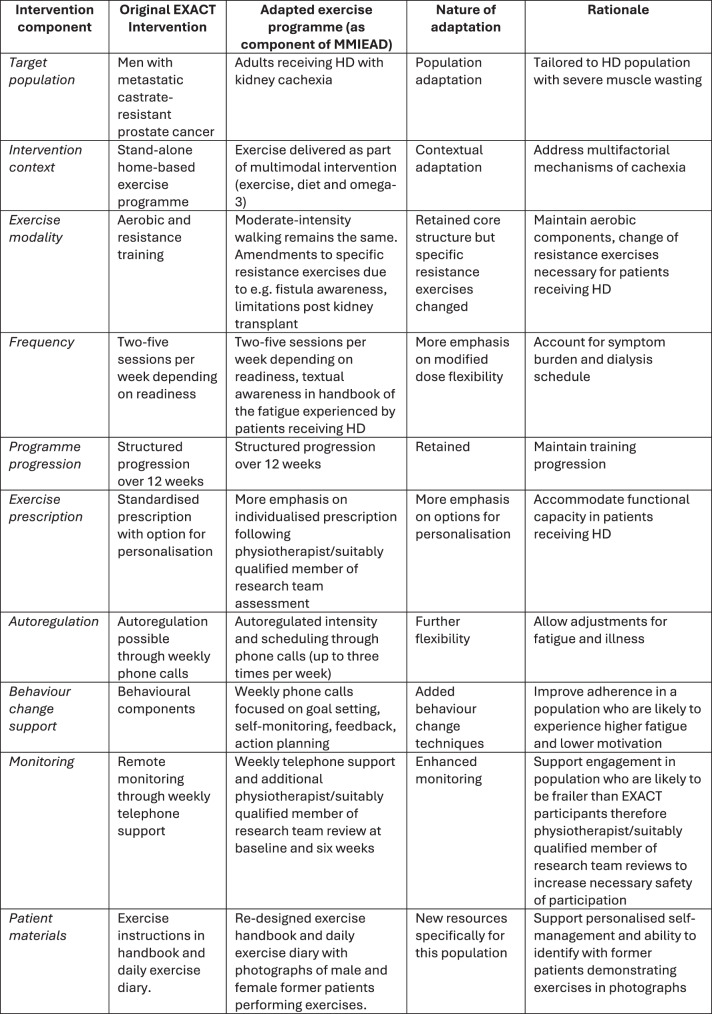
Adaptations made between the EXACT intervention and the new intervention for MMIEAD

**Fig. 3:**
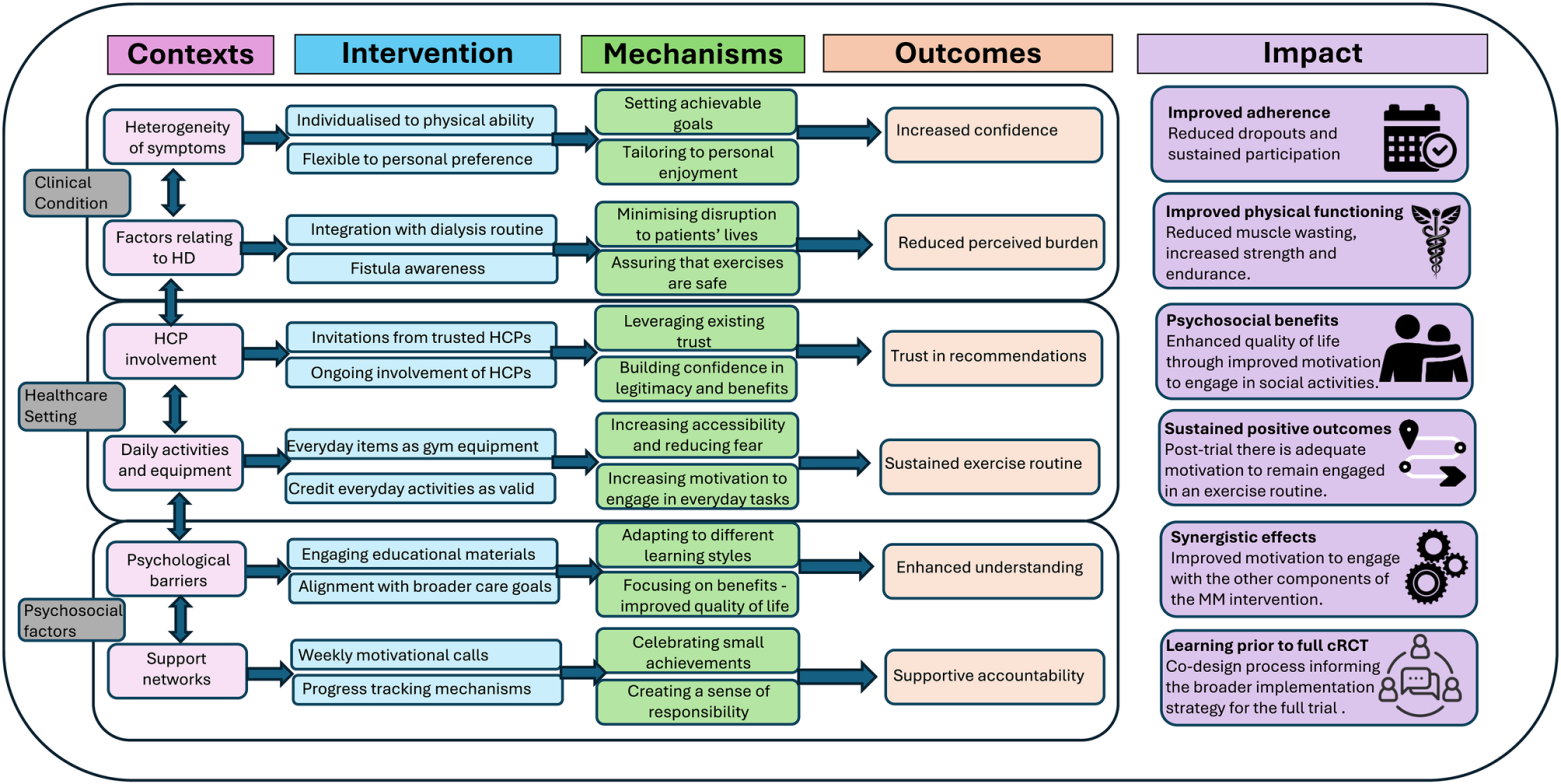
Conceptual model of exercise intervention

A conceptual model was creating a based on necessary changes from the original EXACT intervention (see Fig. 2) which included comparison with existing conceptual models and implementation science literature. Post further consultation with our wider international, multidisciplinary stakeholder group and public co-design partners (November 2024), a final conceptual model was developed (See discussion - Fig. 3). The last stage of the co-design process included developing the associated resources for the exercise intervention in a final workshop with three public involvement representatives and three academic staff with experience in exercise interventions. During this final workshop, the suitability of the amendments to the exercise programme were confirmed by the public co-design partners. Two former patients who had experience of receiving HD performed the amended exercises and consented to photographs being taken. Post workshop, the photographs were integrated into the amended booklet and text subsequently changed reflective of the specific exercises which were deemed suitable. Finally the amendments were checked by the research team and sent to a member of the team who had previously implemented EXACT to ensure agreement.

## Discussion

Following updated Research Council’s (MRC) guidance [50], we have reported on how we collaboratively developed the exercise component of the MMIEAD intervention and prioritised public co-design partners voices [51] usingBird and colleagues [32] Generative Framework for Healthcare Innovation. Local contextual domains with an equity focus [52] have been translated to practical outworking through our adoption of CIMOI [45] to analyse and code data from the workshops to produce our conceptual model (see Fig. 3: Conceptual model of exercise intervention). The conceptual model that we developed describes the features of the context, uses evidence-based key components of the intervention with amendments based on public involvement representatives’ recommendations and details how the mechanisms are anticipated to influence outcomes and subsequent impact. Considering there is a lack of evidence available relating to exercise for renal cachexia we have focused our discussion on the wider literature providing a rationale for personalisation, adding resistance exercise and maintaining the domiciliary setting from the EXACT programme. This is followed by a final rationale for our focus on intervention-context alignment in preparation for integration into the MMIEAD feasibility trial.

### The rationale for personalisation

The certainty of evidence is low regarding the effect of multimodal interventions for cachexia as relating to improvements in physical functioning namely due to the small body of evidence Nevertheless, it is notable that all studies in a recent systematic review [53] were individually tailored and there was evidence of improvements in multiple domains, most notably physical endurance and depression scores. In a relevant Cochrane review [23], authors describe a strong rationale for the use of exercise for cancer cachexia.However, there are no current data from RCTs to elucidate the specific effects nor the specific modalities in this population. In the HD population, the findings of our co-design work align with the majority of factors contributing to sustainable exercise programmes identified by Bennett and colleagues [54]. Although focused on peritoneal dialysis authors [54] describe the need for dedicated exercise professionals, dialysis and medical staff commitment, adequate physical requirements of equipment and space, interesting and stimulating exercise and individual prescription. Although clinical recommendations for people receiving dialysis exist, there is wide variation regarding exercise type, intensity, timing, and safety precautions and there is an absence of directive guidelines [26]. Using our adapted handbook, personalisation will be prioritised and the exercise diary will help to record exercise adherence and therefore demonstrate whether this component of the intervention is feasible.

### The rationale for adding resistance exercise

The PEDAL trial (the PrEscription of intraDialytic exercise to improve quAlity of Life) [55] the CYCLE-HD study [56] and the DiaTT study [57] are three relevant recent large interventional studies which examined the clinical effectiveness of intradialytic cycling. The combined data from the PEDAL trial and CYCLE-HD study demonstrated no impact on body composition with six-month intradialytic cycling exercise training in patients receiving HD [58]. Furthermore, the overall adherence amongst the exercise groups in PEDAL and CYCLE-HD was suboptimal, with more than half (57%) completing less than 70% of the prescribed exercise sessions [58]. The lack of observed effect with PEDAL [55] and CYCLE-HD [56] is in contrast to the DiaTT study [57], which demonstrated that 12 months of intradialytic combined endurance and resistance exercise training significantly improved physical function (60s sit-to-stand test) compared to usual care. Notably the DiaTT study [57] included a longer intervention period (12 months), a progressive resistance training intervention and although the intention was for intradialytic exercise sessions, a significant period was completed at home (due to the pandemic). The DiaTT study [57] therefore introduced telephone consultancy on a weekly basis until intradialytic exercise training could continue. Although it is not entirely clear if the observed effect was influenced by the unprecedented circumstances of the pandemic or whether this is due to the longer duration from best evidence, to date it appears that adding resistance exercise into a home-based programme with added telephone consultations for patients receiving HD has the potential to improve outcomes.

### The rationale for a home-based programme

The choice of place of exercise in this multimodal intervention has been an important decision and although influenced by our public involvement co-design partners we have also aligned with wider patient centred perspectives on the benefits and barriers to engaging in exercise for patients receiving HD. For example, a survey of patients receiving HD (*n* = 423) reported that they wanted to exercise at home (73%) using a combination of aerobic and resistance training (41%), regardless of modality or age category [59]. We have also been influenced by the fact that differing evidence exists on the safety and effectiveness of intradialytic exercise. For example, a systematic review [60] suggests that regardless of modality, exercise interventions in patients receiving HD improved objective measures of physical function. In contrast, a recent meta-analysis [61] and Cochrane review, wherein most exercise interventions were conducted during dialysis [62] concluded that the safety of exercise during dialysis remains uncertain. Although this is most likely due to the adverse effects of exercise training in adults undergoing dialysis being rarely reported and poorly defined [62]. There are also concerns relating to the potential disruption of cardiac haemodynamics [61] which could consequently increase the risk of complications in patients receiving HD. Ultimately there is more consensus that developing safe and effective individualised exercise programmes requires collaboration among a multidisciplinary team [54, 60, 61, 63]. This is what we have strived to do through a comprehensive approach integrating clinical, academic and experiential expertise alongside the valuable voices of those with lived experience to co-design adaptions to the EXACT home-based aerobic and resistance exercise programme.

### Integration into the MMIEAD feasibility trial

To ensure optimum intervention-context alignment we have interwoven the findings from this co-design activity for the exercise component [37] into our larger feasibility trial design. The adoption of PRISM’s context-oriented approach in addition to the co-design work for the exercise component has further helped us to prepare accordingly for the intervention, for example taking into consideration the infrastructure supporting the intervention [37]. Our conceptual model (See Fig. 3) aligns with our Theory of Change (ToC) map [51] for the full multimodal intervention for renal cachexia, and has subsequently provided more detailed intervention plans for the intervention as a whole. Alongside efforts to ensure involvement from practising nephrologists, physiologists, dieticians, clinical research nurses and academics we have actively demonstrated an ongoing and iterative engagement process with public involvement representatives not only through this co-design process but also through ongoing active involvement in the expert reference group for MMIEAD [37]. Public co-design partners priorities, needs, and values have been and continue to be deemed equally important alongside organisational perspectives, resources, and infrastructure because we are aware that these factors impact an intervention’s uptake, equitable implementation, and impact [52].

Using best evidence to date, exercise is likely to be a crucial component in multimodal interventions to help individuals experiencing or at risk of renal cachexia, maintain body weight, and improve strength to enhance their capacity for independent functioning [9–13]. Our hope is that through this feasibility trial and subsequent definitive trial, evidence will be generated for those with or at risk of renal cachexia and research findings will be applied into multimodal interventions which include exercise as a component [64].

### Strengths and limitations

Using CIMOI configuration, we have developed an in-depth understanding of what is needed in the exercise component, how and why it should achieve impact and have designed the processes on best evidence available to achieve the strongest external validity possible [65, 66]. A distinctive strength of this public involvement work is based on having a co-designed conceptual model which provides a rationale for why this exercise component should in theory work. If MMIEAD does not influence the outcomes and cause impact as expected, this conceptual model will help us to ascertain whether the lack of effectiveness of the intervention as a whole is due to the design of the exercise intervention, problems with the implementation or ineffectiveness of the intervention. This component has adopted an iterative design and will continue with agility. Where necessary, amendments will be put in place to recognise and respond to the recruitment profile in each site. The main limitation of this work is based on the weakness that although all participants in the co-design workshops had experience of either receiving HD or caring for a loved one receiving HD, we are unsure whether any had experienced renal cachexia. Due to the low physical functioning of those who have or are at risk of renal cachexia it would have been particularly difficult to recruit workshop attendees, if we had specified this a necessary criterion. Furthermore, if we had specified this criterion, we could not have accurately confirmed eligibility without collecting data unsuitable for co-design activity to make an assessment. To ensure that this limitation did not detract from our co-design activity we designed the first workshop with an educational component on what renal cachexia is and how it can affect physical functioning to ensure awareness of the added physical manifestations of renal cachexia for those receiving HD.

## Conclusion

Using the generative co-design framework for healthcare innovation, a conceptual model has been produced to promote optimal recruitment and adherence of an exercise intervention as part of a multimodal intervention for renal cachexia management in practice. This has informed component design, the wider implementation plan and evaluation design of a multimodal intervention for renal cachexia (MMIEAD) [40]. The results of this study provide the basis for the further design and development of the exercise component as part of the full scale MMIEAD intervention for those with or at risk of renal cachexia. The model’s emphasis on personalisation, resistance exercise and the preference of domiciliary environment in particular add to the literature on preferred exercise interventions for those receiving HD. In the following phase, we will test and possibly further adapt the conceptual model through the findings of this feasibility cRCT in preparation for the full-scale trial.

## Electronic supplementary material

Below is the link to the electronic supplementary material.

Supplementary material 1

Supplementary material 2

## Data Availability

All information is included in the manuscript.

## Abbreviations

CKD: Chronic kidney disease
CIMOI: Context-intervention-mechanism-outcome-impact
Crct: Cluster randomised controlled trial
HD: Haemodialysis
HCPs: Healthcare professionals
HSC: Health and Social Care
HR-QoL: Health related quality of life
KDOQI: Kidney Disease Outcomes Quality Initiative
MRC: Medical Research Council
MMIEAD: Multi-Modal Integrated intervention combining Exercise, Anti-inflammatory & Dietary advice
NHS: National Health Service
NIHR: National Institute of Health Research
NIKPA: Northern Ireland Kidney Patients Association
NIKRF: Northern Ireland Kidney Research Fund
PRISM: Practical, Robust Implementation and Sustainability Model
PT: Personal Trainer
QoL: Quality of life
QUB: Queen’s University Belfast
RCT: Randomised controlled trial
ToC: Theory of change
UK: United Kingdom
UCL: University College London

## References

[1] von Haehling S, Anker MS, Anker SD. Prevalence and clinical impact of cachexia in chronic illness in Europe, USA, and Japan: facts and numbers update 2016. Wiley Online Library; 2016. 507–09. 10.1002/jcsm.12167 PMC5114624 27891294

[2] Koppe L, Fouque D, Kalantar-Zadeh K. Kidney cachexia or protein-energy wasting in chronic kidney disease: facts and numbers. J Cachexia, Sarcopenia Muscle. 2019;10(3):479–84. 10.1002/jcsm.12421. 30977979 10.1002/jcsm.12421 PMC6596400

[3] Sanchez-Rodriguez D, Annweiler C, Cederholm T. A translational approach for the clinical application of recently updated definitions of malnutrition (GLIM) and sarcopenia (EWGSOP2). Maturitas. 2019;122:89–90. 10.1016/j.maturitas.2018.11.013. 30497786 10.1016/j.maturitas.2018.11.013

[4] Aversa Z, Costelli P, Muscaritoli M. Cancer-induced muscle wasting: latest findings in prevention and treatment. Ther Adv Med Oncol. 2017;9(5):369–82. 10.1177/1758834017698643. 28529552 10.1177/1758834017698643 PMC5424865

[5] Slee A, Reid J. Exercise and nutrition interventions for renal cachexia. Curr Opin Clin Nutr Metab Care. 2024;27(3):219–25. 10.1097/MCO.0000000000001022. 38386361 10.1097/MCO.0000000000001022 PMC10990023

[6] Crawford J. What are the criteria for response to cachexia treatment? Ann Palliat Med. 2019;8(1):43–49. 30685983 10.21037/apm.2018.12.08

[7] Reid J, Blair C, Dempster M, McKeaveney C, Slee A, Fitzsimons D. Multimodal interventions for cachexia management. Cochrane Database Syst Rev. 2025;3(3): CD015749. 10.1002/14651858.CD015749.pub2. 10.1002/14651858.CD015749.pub2 PMC11934851 40130780

[8] McKeaveney C, Maxwell P, Noble H, Reid J. A critical review of multimodal interventions for cachexia. Adv Nutr. 2021;12(2):523–32. 10.1093/advances/nmaa111. 32970097 10.1093/advances/nmaa111 PMC8262513

[9] Kalantar-Zadeh K, Moore LW. Improving muscle strength and preventing Sarcopenia and cachexia in chronic Kidney disease and transplanted patients by physical activity and exercise. J Ren Nutr. 2019;29(6):465–66. 10.1053/j.jrn.2019.09.005. 31676147 10.1053/j.jrn.2019.09.005

[10] Wilkinson TJ, Shur NF, Smith AC. “Exercise as medicine” in chronic kidney disease. Scand J Med Sci Sports. 2016;26(8):985–88. 10.1111/sms.12714 27334146 10.1111/sms.12714

[11] Anker SD, Morley JE. Cachexia: a nutritional syndrome? J Cachexia, Sarcopenia Muscle. 2015;6(4):269–71. 10.1002/jcsm.12088. 26675043 10.1002/jcsm.12088 PMC4670732

[12] Watson EL, Kosmadakis GC, Smith AC, Viana JL, Brown JR, Molyneux K, et al. Combined walking exercise and alkali therapy in patients with CKD4-5 regulates intramuscular free amino acid pools and ubiquitin E3 ligase expression. Eur J Appl Physiol. 2013;113(8):2111–24. 10.1007/s00421-013-2628-5. 23591985 10.1007/s00421-013-2628-5

[13] Rahbar Saadat Y, Abbasi A, Hejazian SS, Hekmatshoar Y, Ardalan M, Farnood F, et al. Combating chronic kidney disease-associated cachexia: a literature review of recent therapeutic approaches. BMC Nephrol. 2025;26(1):133. 10.1186/s12882-025-04057-8. 40069669 10.1186/s12882-025-04057-8 PMC11895341

[14] Yoon M-S. mTOR as a key regulator in maintaining skeletal muscle mass. Frontiers in physiology. 2017 8:788. 10.3389/fphys.2017.00788 PMC5650960 29089899

[15] Moura SRG, Correa HL, Neves RVP, Santos CAR, Neto LSS, Silva VL, et al. Effects of resistance training on hepcidin levels and iron bioavailability in older individuals with end-stage renal disease: a randomized controlled trial. Exp Gerontol. 2020;139:111017. 10.1016/j.exger.2020.111017. 32634551 10.1016/j.exger.2020.111017

[16] Rubio-Ruiz ME, Guarner-Lans V, Perez-Torres I, Soto ME. Mechanisms underlying metabolic syndrome-related Sarcopenia and possible therapeutic measures. Int J Mol Sci. 2019;20(3):647. 10.3390/ijms20030647. 30717377 10.3390/ijms20030647 PMC6387003

[17] McKeaveney C, Slee A, Adamson G, Davenport A, Farrington K, Fouque D, et al. Using a generic definition of cachexia in patients with kidney disease receiving haemodialysis: a longitudinal (pilot) study. Nephrol Dial Transpl. 2021;36(10):1919–26. 10.1093/ndt/gfaa174. 10.1093/ndt/gfaa174 33150449

[18] Arends J, Strasser F, Gonella S, Solheim TS, Madeddu C, Ravasco P, et al. Cancer cachexia in adult patients: ESMO clinical practice Guidelines(☆). ESMO Open. 2021;6(3):100092. 10.1016/j.esmoop.2021.100092. 34144781 10.1016/j.esmoop.2021.100092 PMC8233663

[19] Stevens PE, Ahmed SB, Carrero JJ, Foster B, Francis A, Hall RK, et al. KDIGO, 2024 clinical practice guideline for the evaluation and management of chronic kidney disease. Kidney Int. 2024;105(4):S117–314. 10.1016/j.kint.2023.10.018 10.1016/j.kint.2023.10.018 38490803

[20] Taryana AA, Krishnasamy R, Bohm C, Palmer SC, Wiebe N, Boudville N, et al. Physical activity for people with chronic kidney disease: an international survey of nephrologist practice patterns and research priorities. BMJ Open. 2019;9(12):e032322. 10.1136/bmjopen-2019-032322. 10.1136/bmjopen-2019-032322 PMC6936996 31857307

[21] Clyne N, Cupisti A, Grupp C, Kouidi E, Segura-Orti E, Provenzano PF, et al. Lack of physiotherapy resources restricts exercise prescription for patients with chronic kidney disease-the EUropean SUrvey on REnal EXercise (EUSUREX). Clin Kidney J. 2025;18(11):sfaf291. 10.1093/ckj/sfaf291. 10.1093/ckj/sfaf291 PMC12596180 41215781

[22] De Brandt J, Spruit MA, Derave W, Hansen D, Vanfleteren LE, Burtin C. Changes in structural and metabolic muscle characteristics following exercise-based interventions in patients with COPD: a systematic review. Expert Rev Respir Med. 2016;10(5):521–45. 10.1586/17476348.2016.1157472. 26901573 10.1586/17476348.2016.1157472

[23] Grande AJ, Silva V, Sawaris Neto L, Teixeira Basmage JP, Peccin MS, Maddocks M. Exercise for cancer cachexia in adults. Cochrane Database Syst Rev. 2021;3(3):Cd010804. 10.1002/14651858.CD010804.pub3. 10.1002/14651858.CD010804.pub3 PMC8094916 33735441

[24] Hristea D, Deschamps T, Paris A, Lefrancois G, Collet V, Savoiu C, et al. Combining intra-dialytic exercise and nutritional supplementation in malnourished older haemodialysis patients: towards better quality of life and autonomy. Nephrol (Carlton). 2016;21(9):785–90. 10.1111/nep.12752. 10.1111/nep.12752 26890997

[25] Solheim TS, Laird BJ, Balstad TR, Stene GB, Baracos V, Bye A, et al. Results from a randomised, open-label trial of a multimodal intervention (exercise, nutrition and anti-inflammatory medication) plus standard care versus standard care alone to attenuate cachexia in patients with advanced cancer undergoing chemotherapy. Am Soc Clin Oncol. 2024.

[26] Lambert K, Lightfoot CJ, Jegatheesan DK, Gabrys I, Bennett PN. Physical activity and exercise recommendations for people receiving dialysis: a scoping review. PLoS One. 2022;17(4):e0267290. 10.1371/journal.pone.0267290. 10.1371/journal.pone.0267290 PMC9049336 35482797

[27] Huang M, Lv A, Wang J, Xu N, Ma G, Zhai Z, et al. Exercise training and outcomes in Hemodialysis patients: systematic review and Meta-analysis. Am J Nephrol. 2019;50(4):240–54. 10.1159/000502447. 31454822 10.1159/000502447

[28] Greenwood SA, Young HML, Briggs J, Castle EM, Walklin C, Haggis L, et al. Evaluating the effect of a digital health intervention to enhance physical activity in people with chronic kidney disease (Kidney BEAM): a multicentre, randomised controlled trial in the UK. Lancet Digit Health. 2024;6(1):e23–32. 10.1016/S2589-7500(23)00204-2. 10.1016/S2589-7500(23)00204-2 37968170

[29] Jhamb M, Devaraj SM, Alemairi M, Lavenburg LM, Shiva S, Yabes JG, et al. A comprehensive exercise (COMEX) intervention to optimize exercise participation for improving patient-centered outcomes and physical functioning in patients receiving Hemodialysis: development and pilot testing. Kidney Med. 2023;5(11):100720. 10.1016/j.xkme.2023.100720. 37928754 10.1016/j.xkme.2023.100720 PMC10623365

[30] Mayes J, Billany RE, Vadaszy N, Young HML, Castle EM, Bishop NC, et al. The rapid development of a novel kidney-specific digital intervention for self-management of physical activity and emotional well-being during the COVID-19 pandemic and beyond: kidney Beam. Clin Kidney J. 2022;15(3):571–73. 10.1093/ckj/sfab239. 35198162 10.1093/ckj/sfab239 PMC8690269

[31] Greenhalgh T, Hinton L, Finlay T, Macfarlane A, Fahy N, Clyde B, et al. Frameworks for supporting patient and public involvement in research: systematic review and co-design pilot. Health Expect. 2019;22(4):785–801. 10.1111/hex.12888. 31012259 10.1111/hex.12888 PMC6737756

[32] Bird M, McGillion M, Chambers EM, Dix J, Fajardo CJ, Gilmour M, et al. A generative co-design framework for healthcare innovation: development and application of an end-user engagement framework. Res Involv Engagem. 2021;7(1):12. 10.1186/s40900-021-00252-7. 33648588 10.1186/s40900-021-00252-7 PMC7923456

[33] Batalden M, Batalden P, Margolis P, Seid M, Armstrong G, Opipari-Arrigan L, et al. Coproduction of healthcare service. BMJ Qual Saf. 2016;25(7):509–17. 10.1136/bmjqs-2015-004315. 26376674 10.1136/bmjqs-2015-004315 PMC4941163

[34] Donetto S, Pierri P, Tsianakas V, Robert G. Experience-based Co-design and healthcare improvement: realizing Participatory design in the public sector. Des J. 2015;18(2):227–48. 10.2752/175630615X14212498964312.

[35] Slattery P, Saeri AK, Bragge P. Research co-design in health: a rapid overview of reviews. Health Res Policy Syst. 2020;18(1):17. 10.1186/s12961-020-0528-9. 32046728 10.1186/s12961-020-0528-9 PMC7014755

[36] Jolles MP, Fort MP, Glasgow RE. Aligning the planning, development, and implementation of complex interventions to local contexts with an equity focus: application of the PRISM/RE-AIM framework. Int J Equity Health. 2024;23(1):41. 10.1186/s12939-024-02130-6. 10.1186/s12939-024-02130-6 PMC10898074 38408990

[37] Baum F, MacDougall C, Smith D. Participatory action research. J Epidemiol Community Health. 2006;60(10):854–57. 10.1136/jech.2004.028662. 16973531 10.1136/jech.2004.028662 PMC2566051

[38] Viswanathan M, Ammerman A, Eng E, Garlehner G, Lohr KN, Griffith D, et al. Community-based participatory research: assessing the evidence: summary. AHRQ Evidence Report Summaries. 2004. PMC4780908 15460504

[39] Roberts LW. What is community-based participatory research? Community-based Participatory research for improved mental healthcare: a manual for clinicians and researchers. Springer; 2012. p. 1–9.

[40] Reid J, Blair C, Slee A, McKeaveney C, Maxwell AP, Adell V, et al. Multi-modal integrated intervention combining exercise, anti-inflammatory, and dietary advice (MMIEAD) for adults with kidney cachexia: protocol for a mixed-methods feasibility cluster randomised controlled trial and process evaluation. Pilot Feasibility Stud. 2026. 10.1186/s40814-026-01784-z42. 10.1186/s40814-026-01784-z PMC13020271 41715172

[41] Boyd H, McKernon S, Mullin B, Old A. Improving healthcare through the use of co-design. N Z Med J. 2012;125(1357):76–87. PMID: 22854362. 22854362

[42] Iniesto F, Littlejohn A, Charitonos K. A review of research with co-design methods in health education. Open Educ Stud. 2022. 10.1515/edu-2022-0017

[43] Frank L, Basch E, Selby JV. Patient-centered outcomes research I. The PCORI perspective on patient-centered outcomes research. JAMA. 2014;312(15):1513–14. 10.1001/jama.2014.11100. 25167382 10.1001/jama.2014.11100

[44] Robert G, Locock L, Williams O, Cornwell J, Donetto S, Goodrich J. Co-producing and co-designing. Cambridge University Press; 2022.

[45] Denyer D, Tranfield D, van Aken JE. Developing design propositions through research synthesis. Organ Stud. 2008;29(3):393–413. 10.1177/0170840607088020.

[46] Brown M, Murphy MH, McAneney H, McBride K, Crawford F, Cole A, et al. Feasibility of home-based exercise training during adjuvant treatment for metastatic castrate-resistant prostate cancer patients treated with an androgen receptor pathway inhibitor (EXACT). Support Care Cancer. 2023;31(7):442. 10.1007/s00520-023-07894-1. 37402060 10.1007/s00520-023-07894-1 PMC10319656

[47] Manfredini F, Mallamaci F, D’Arrigo G, Baggetta R, Bolignano D, Torino C, et al. Exercise in patients on dialysis: a multicenter, randomized clinical trial. J Am Soc Nephrol. 2017;28(4):1259–68. 10.1681/asn.2016030378. 27909047 10.1681/ASN.2016030378 PMC5373448

[48] Mallamaci F, D’Arrigo G, Tripepi G, Lamberti N, Torino C, Manfredini F, et al. Long-Term effect of physical exercise on the risk for hospitalization and death in dialysis patients: a post-trial Long-Term observational study. Clin J Am Soc Nephrol. 2022;17(8):1176–82. 10.2215/cjn.03160322. 35878932 10.2215/CJN.03160322 PMC9435990

[49] Noor H, Reid J, Slee A. Resistance exercise and nutritional interventions for augmenting sarcopenia outcomes in chronic kidney disease: a narrative review. J Cachexia, Sarcopenia Muscle. 2021;12(6):1621–40. 10.1002/jcsm.12791. 34585539 10.1002/jcsm.12791 PMC8718072

[50] Skivington K, Matthews L, Simpson SA, Craig P, Baird J, Blazeby JM, et al. A new framework for developing and evaluating complex interventions: update of Medical research Council guidance. BMJ. 2021;374:n2061. 10.1136/bmj.n2061. 10.1136/bmj.n2061 PMC8482308 34593508

[51] Blair C, Slee A, Davenport A, Fouque D, Johnston W, Kalantar-Zadeh K, et al. editors. Developing an evidence and theory based multimodal integrative intervention for the management of renal cachexia: a theory of change. In: Healthcare. Multidisciplinary Digital Publishing Institute; 2022. 10.3390/healthcare10122344 PMC9777598 36553868

[52] Bauer MS, Kirchner J. Implementation science: what is it and why should I care? Psychiatry Res. 2020;283:112376. 10.1016/j.psychres.2019.04.025. 31036287 10.1016/j.psychres.2019.04.025

[53] Hall CC, Cook J, Maddocks M, Skipworth RJE, Fallon M, Laird BJ. Combined exercise and nutritional rehabilitation in outpatients with incurable cancer: a systematic review. Support Care Cancer. 2019;27(7):2371–84. 10.1007/s00520-019-04749-6. 30944994 10.1007/s00520-019-04749-6 PMC6541700

[54] Bennett PN, Bohm C, Harasemiw O, Brown L, Gabrys I, Jegatheesan D, et al. Physical activity and exercise in peritoneal dialysis: International Society for peritoneal dialysis and the global renal exercise Network practice recommendations. Perit Dial Int. 2022;42(1):8–24. 10.1177/08968608211055290. 34743628 10.1177/08968608211055290

[55] Greenwood SA, Koufaki P, Macdonald JH, Bhandari S, Burton JO, Dasgupta I, et al. Randomized trial-PrEscription of intraDialytic exercise to improve quAlity of life in patients receiving Hemodialysis. Kidney Int Rep. 2021;6(8):2159–70. 10.1016/j.ekir.2021.05.034. 34386665 10.1016/j.ekir.2021.05.034 PMC8343798

[56] Graham-Brown MPM, March DS, Young R, Highton PJ, Young HML, Churchward DR, et al. A randomized controlled trial to investigate the effects of intra-dialytic cycling on left ventricular mass. Kidney Int. 2021;99(6):1478–86. 10.1016/j.kint.2021.02.027. 34023029 10.1016/j.kint.2021.02.027

[57] Anding-Rost K, von Gersdorff G, von Korn P, Ihorst G, Josef A, Kaufmann M, et al. Exercise during Hemodialysis in patients with chronic Kidney failure. NEJM Evid. 2023;2(9):EVIDoa2300057. 10.1056/EVIDoa2300057. 10.1056/EVIDoa2300057 38320198

[58] Ng KP, Macdonald JH, Young R, March DS, Graham-Brown MPM, Mercer TH, et al. Body composition and intradialytic exercise in kidney disease: a combined analysis of the PEDAL and CYCLE-HD randomised controlled trials. J Cachexia, Sarcopenia Muscle. 2025;16(2):e13748. 10.1002/jcsm.13748. 10.1002/jcsm.13748 PMC11873537 40026059

[59] Moorman D, Suri R, Hiremath S, Jegatheswaran J, Kumar T, Bugeja A, et al. Benefits and Barriers to and desired outcomes with exercise in patients with ESKD. Clin J Am Soc Nephrol. 2019;14(2):268–76. 10.2215/CJN.09700818. 30696660 10.2215/CJN.09700818 PMC6390914

[60] Clarkson MJ, Bennett PN, Fraser SF, Warmington SA. Exercise interventions for improving objective physical function in patients with end-stage kidney disease on dialysis: a systematic review and meta-analysis. Am J Physiol Renal Physiol. 2019;316(5):F856–72. 10.1152/ajprenal.00317.2018. 10.1152/ajprenal.00317.2018 30759022

[61] Liu H, Zhang M, Chen M, Chen L, Huang T, Ye Z. Meta-analysis of the effects of exercise interventions on dialysis patients with cardiac function disorders. Front Med (Lausanne). 2025;12:1573498. 10.3389/fmed.2025.1573498. 40432722 10.3389/fmed.2025.1573498 PMC12106465

[62] Bernier-Jean A, Beruni NA, Bondonno NP, Williams G, Teixeira-Pinto A, Craig JC, et al. Exercise training for adults undergoing maintenance dialysis. Cochrane Database Syst Rev. 2022;1(1):Cd014653. 10.1002/14651858.Cd014653. 10.1002/14651858.CD014653 PMC8752366 35018639

[63] Li T, Lv A, Xu N, Huang M, Su Y, Zhang B, et al. Barriers and facilitators to exercise in haemodialysis patients: a systematic review of qualitative studies. J Adv Nurs. 2021;77(12):4679–92. 10.1111/jan.14960. 34258784 10.1111/jan.14960

[64] Fearon K, Argiles JM, Baracos VE, Bernabei R, Coats A, Crawford J, et al. Request for regulatory guidance for cancer cachexia intervention trials. J Cachexia, Sarcopenia Muscle. 2015;6(4):272–74. 10.1002/jcsm.12083. 26675232 10.1002/jcsm.12083 PMC4670733

[65] Ward S, Donovan HS, Serlin RC. An alternative view on “an alternative paradigm”. Res Nurs Health. 2003;26(3):256. PMCID: PMC2526466 NIHMSID: NIHMS63468 PMID: 12754733. 12754733 10.1002/nur.10088 PMC2526466

[66] De Silva MJ, Breuer E, Lee L, Asher L, Chowdhary N, Lund C, et al. Theory of change: a theory-driven approach to enhance the medical research council’s framework for complex interventions. Trials. 2014;15:267. 10.1186/1745-6215-15-267. 24996765 10.1186/1745-6215-15-267 PMC4227087

