# Supplementary material for: Using a generative co-design framework to adapt an exercise intervention as part of a multimodal intervention for patients’ receiving haemodialysis with or at risk of renal cachexia
Source: Res Involv Engagem. 2026 Apr 17;12:73. doi: 10.1186/s40900-026-00875-8 (PMC13214384; doi:10.1186/s40900-026-00875-8)
Supplement: Supplementary file 1 — Supplementary material 1 [file 40900_2026_875_MOESM1_ESM.docx]

Supplementary document 2: GRIPP2 short form

| Section and topic | Item |
| --- | --- |
| 1: Aim | This study was developed with advice and contribution from patient and carer representatives from Northern Ireland Kidney Patients Association - NIKPA (public co-design partners). One of these individuals have been involved in the design of the Multi-Modal Integrated Intervention Combining Exercise, Anti-inflammatory & Dietary Advice (MMIEAD) for Kidney Cachexia from inception (Fez Awan) who is a co-author which reflects his ongoing involvement at all stages of the research process. The aim of involvement was to help ensure the exercise component of the MMIEAD intervention addressed the priorities and needs of patients who will be recruited into the study. |
| 2: Methods | Our public co-design partners played an active role in shaping the methodology of this project. We maintained regular contact with them throughout the co-design process, engaging through three workshops as well as ongoing communication by phone, email and MS Teams meetings. |
| 3: Study results | Primary results and dissemination plan were discussed at the second and third workshops. Our public co-design partners assessed the relevance of the initial findings from the feedback at the second workshop and helped the team to develop the conceptual model (figure 1 in manuscript). |
| 4: Discussion and conclusions | The input of our public so-design partners helped to shape the discussion and conclusion to be more relevant and practical, making this exercise component of the MMIEAD intervention more applicable to those it aimed to help. |
| 5: Reflections/critical perspective | Our public so-design partners from NIKPA have worked with our research team over a period of years. The team's relationship with our partners have strengthened over the years, highlighting the importance of sustaining long-term collaborations with patient and public involvement partners.  There were limitations to our public so-design partners involved in this work. Considering the larger group (excluding Fez Awan) are not involved in the MMIEAD expert reference group we did not invite comments directly from then regarding this manuscript, therefore, did not include them as co-authors. However, we have ensured that we have acknowledged their much appreciated, input in the acknowledgements section. |
