# Supplementary material for: Using a generative co-design framework to adapt an exercise intervention as part of a multimodal intervention for patients’ receiving haemodialysis with or at risk of renal cachexia
Source: Res Involv Engagem. 2026 Apr 17;12:73. doi: 10.1186/s40900-026-00875-8 (PMC13214384; doi:10.1186/s40900-026-00875-8)
Supplement: Supplementary file 2 — Supplementary material 2 [file 40900_2026_875_MOESM2_ESM.docx]

Supplementary document 1

Table 1: Attendance at workshop 1

| **Public co-design participants /Academic** | **Country** | **Gender** | **Relevance** | **Affiliation** |
| --- | --- | --- | --- | --- |
| Public co-design participant | England | Female | Experience of HD | Kidney Care UK |
| Public co-design participant | England | Male | Experience of HD | Kidney Care UK |
| Public co-design participant | Northen Ireland | Male | Experience of HD | NIKPA |
| Public co-design participant | Northen Ireland | Female | Former carer for family member receiving HD | NIKPA |
| Public co-design participant | Northen Ireland | Female | Former carer for family member receiving HD | NIKRF |
| Public co-design participant | Northen Ireland | Male | Experience of HD | NIKPA |
| Public co-design participant | Northen Ireland | Male | Experience of HD | NIKPA |
| Public co-design participant | Northen Ireland | Female | Experience of HD | NIKPA |
| Academic | England | Male | Experience in designing exercise interventions, specialism in renal cachexia. PT and exercise referral specialist. | UCL |
| Academic | Northen Ireland | Female | Experience in designing exercise interventions in chronic illness, specialism in renal cachexia. | QUB |
| Academic | Northen Ireland | Female | Experience in designing exercise interventions, specialism in renal cachexia. PT and exercise referral specialist. | QUB |
| Key: Haemodialysis (HD), Northern Ireland Kidney Patients Association (NIKPA), Northern Ireland Kidney Research Fund (NIKRF), Personal Trainer (PT), Queen’s University Belfast (QUB), United Kingdom (UK), University College London (UCL) | | | | |

Table 2: Attendance at workshop 2

| **Public co-design participant/academic** | **Country** | **Gender** | **Relevance** | **Affiliation** |
| --- | --- | --- | --- | --- |
| Public co-design participant | England | Male | Experience of HD | Kidney Care UK |
| Public co-design participant | Northen Ireland | Male | Experience of HD | NIKPA |
| Public co-design participant | Northen Ireland | Female | Carer for family member receiving HD | NIKPA |
| Public co-design participant | Northen Ireland | Female | Former carer for family member receiving HD | NIKRF |
| Public co-design participant | Northen Ireland | Male | Experience of HD | NIKPA |
| Public co-design participant | Northen Ireland | Female | Former carer for family member receiving HD | NIKPA |
| Academic | England | Male | Professor in Sport and Exercise Science. Experience in designing exercise interventions in chronic illness, specialism in renal cachexia. | University of Greenwich |
| Academic | England | Male | Experience in designing exercise interventions, specialism in renal cachexia. PT and exercise referral specialist. | UCL |
| Academic | Northen Ireland | Female | Experience in designing exercise interventions, specialism in renal cachexia. | QUB |
| Academic | Northen Ireland | Female | Experience in designing exercise interventions in chronic illness, specialism in renal cachexia. | QUB |
| Academic | Northen Ireland | Female | Experience in designing exercise interventions, specialism in renal cachexia. PT and exercise referral specialist. | QUB |
| Key: Haemodialysis (HD), Northern Ireland Kidney Patients Association (NIKPA), Northern Ireland Kidney Research Fund (NIKRF), Personal Trainer (PT), Queen’s University Belfast (QUB), United Kingdom (UK), University College London (UCL) | | | | |

Table 3: Attendance at workshop 3

| **Public co-design participant/Academic** | **Country** | **Gender** | **Relevance** | **Affiliation** |
| --- | --- | --- | --- | --- |
| Public co-design participant | Northen Ireland | Male | Experience of HD | NIKPA |
| Public co-design participant | Northen Ireland | Male | Experience of HD | NIKPA |
| Public co-design participant | Northen Ireland | Female | Former carer for family member receiving HD | NIKPA |
| Academic | England | Male | Experience in designing exercise interventions, specialism in renal cachexia. PT and exercise referral specialist. | UCL |
| Academic | Northen Ireland | Female | Experience in designing exercise interventions in chronic illness, specialism in renal cachexia. | QUB |
| Academic | Northen Ireland | Female | Experience in designing exercise interventions, specialism in renal cachexia. PT and exercise referral specialist. | QUB |
| Key: Haemodialysis (HD), Northern Ireland Kidney Patients Association (NIKPA), Personal Trainer (PT), Queen’s University Belfast (QUB), United Kingdom (UK), University College London (UCL) | | | | |
